# Supplementary material for: The Covid-19 pandemic and mental health of first-year college students: Examining the effect of Covid-19 stressors using longitudinal data
Source: PLoS One. 2021 Mar 5;16(3):e0247999. doi: 10.1371/journal.pone.0247999 (PMC7935268; doi:10.1371/journal.pone.0247999)
Supplement: S1 Table — (PDF) [file pone.0247999.s001.pdf]

S1 Table. Ordinary least squares (OLS) regression estimates for levels of and changes in severity of anxiety and depression symptoms among the Transitions Study longitudinal sample (N=419).

	Anxiety (GAD-7)		Depression (PHQ-8)	
	Symptoms	Changes in Symptoms	Symptoms	Changes in Symptoms
	OLS	OLS	OLS	OLS
	Beta (s.e.)[a]	Beta (s.e.)[a]	Beta (s.e.)[a]	Beta (s.e.)[a]
Employed (Wave I)	-0.231**	-0.226*	-0.280***	-0.222*
	0.109	0.137	0.098	0.120
x Work reduction (student)	0.187	0.226	0.202*	0.160
	0.126	0.157	0.122	0.143
Work reduction (parent)	0.010	0.030	0.114	0.143
	0.089	0.105	0.082	0.099
Distanced learning	0.205***	0.083	0.190***	0.100**
	0.053	0.056	0.045	0.051
Education technology	0.017	0.008	-0.027	-0.009
	0.042	0.047	0.040	0.044
Covid-19 diagnosis	0.098	0.057	0.052	-0.031
	0.102	0.122	0.095	0.107
Covid-19 hospitalization	0.110	0.195	0.005	0.067
	0.154	0.181	0.169	0.200
Social isolation (Wave II)	0.605***	0.463***	0.629***	0.469***
	0.102	0.127	0.100	0.111
Mental health (Wave I)	0.358***		0.424***	
	0.060		0.060	
Social isolation (Wave I)	-0.278**	-0.501***	-0.122	-0.484***
	0.117	0.145	0.124	0.149
R^2	0.457	0.142	0.534	0.166
Joint significance of Covid-19 Stressors				
p-value	0.001	0.254	0.000	0.123

[a]Abbreviations: standard errors, s.e.

Note: All models control for students' psychological resources and social support measures listed in Table 1, missing indicators for psychological resources and social support measures, race, gender, Sexual/gender minority identity, first-generation college student status, age, free/reduced priced-lunch, the week in which the student responded to Wave II, and a constant.

*** $p < 0.001$, ** $p < 0.05$, * $p < 0.1$
