# Supplementary material for: The Covid-19 pandemic and mental health of first-year college students: Examining the effect of Covid-19 stressors using longitudinal data
Source: PLoS One. 2021 Mar 5;16(3):e0247999. doi: 10.1371/journal.pone.0247999 (PMC7935268; doi:10.1371/journal.pone.0247999)
Supplement: Attachment — Submitted filename: Comments for PONE-D-20-40119.docx [file pone.0247999.s002.docx]

Overall, this article showed an interesting topic as it is important to see the changes anxiety level among the students pre and post-pandemic. However, there were several parts need to be clarified as follows:

1. In the background section, the authors mentioned that the first-year students is critical for academic success. What does it mean? What makes it critical?
2. In table 1, the authors listed the demographic characteristic among the two samples (cross-sectional and longitudinal). Why the authors did not include the family income as it mentioned earlier in the background as the related factors.
3. Table 5 looks not clear. Why did the author provide three marginal effect with different values? Why did the sample in column 3,4,7,8 are different? What makes the difference? How did the authors deal with this issue?
4. Still in table 5, why the authors were not able to calculate the odds ratio as this is important to find the likelihood to have high anxiety?
5. On page 18-19, the authors mentioned we found that students who were already experiencing mental health problems pre-pandemic were at greater odds of experiencing severe symptoms mid-pandemic.

Minor comments:

Period should be placed after the citation. Please see the guideline.
