# Supplementary material for: The Covid-19 pandemic and mental health of first-year college students: Examining the effect of Covid-19 stressors using longitudinal data
Source: PLoS One. 2021 Mar 5;16(3):e0247999. doi: 10.1371/journal.pone.0247999 (PMC7935268; doi:10.1371/journal.pone.0247999)
Supplement: Attachment — Submitted filename: Response to Reviewers.docx [file pone.0247999.s003.docx]

Dear Editor,

Thank you for the opportunity to submit a revised version of The Covid-19 Pandemic and Mental Health of First-Year College Students: Examining the Effect of Covid-19 Stressors Using Longitudinal Data” for your consider for publication as a *research article* in PLOS ONE.

We include below our responses to each of your comments and to those of the referee. We put our responses in italics.

*We have incorporated the references you mentioned in the text on lines 150-162. They are listed here with their reference number:*

(26) Pramukti, I., Strong, C., Sitthimongkol, Y., Setiawan, A., Pandin M. G. R., Yen, C.-F., Lin, C.-Y., Griffiths, M. D., Ko, N.-Y. (2020). Anxiety and suicidal thoughts during the COVID-19 pandemic: A cross-country comparison among Indonesian, Taiwanese, and Thai university students. Journal of Medical Internet Research, 22(12), e24487.
(30) Nathiya D, Singh P, Suman S, Raj P, Tomar BS. Mental health problems and impact on youth minds during the COVID-19 outbreak: Cross-sectional (RED-COVID) survey. Soc Health Behav 2020;3:83-8
(29) Akdeniz G, Kavakci M, Gozugok M, Yalcinkaya S, Kucukay A, Sahutogullari B. A survey of attitudes, anxiety Status, and protective behaviors of the university students during the COVID-19 outbreak in Turkey. Front Psychiatry 2020;11:695

*We have addressed the style requirements as requested in your letter. In formatting the tables to meet the style requirement, we divided Table 5 from our previous submission, into two tables (now Tables 5 and 6) to better fit in the document.*

*We also include the following prompts and our responses with data availability:*

a) If there are ethical or legal restrictions on sharing a de-identified data set, please explain them in detail (e.g., data contain potentially sensitive information, data are owned by a third-party organization, etc.) and who has imposed them (e.g., an ethics committee). Please also provide contact information for a data access committee, ethics committee, or other institutional body to which data requests may be sent.

*The UNC ethics committee has deemed that the data contain potentially sensitive information and that there is a possibility of deductive disclosure, so that the human subjects approval does not allow us to share the de-identified data. The de-identified data will be made available upon request to the Deputy Director of Research at the UNC Carolina Population Center (TransitionsDataRequest@office.unc.edu) with an appropriate restricted use data agreement in place.*

*We have also added the supporting information captions at the end of the text and updated our Supporting Information files and in-text citations in accordance with the journal guidelines.*

Please let us know if there are any other questions or concerns we can address.

Sincerely,

Jane Fruehwirth, Siddartha Biswas and Krista Perreira

Referee Comments with Responses in Italics

*Thank you for taking the time to review our article and for the improvements you suggest.*

Overall, this article showed an interesting topic as it is important to see the changes anxiety level among the students pre and post-pandemic. However, there were several parts need to be clarified as follows:

1. In the background section, the authors mentioned that the first-year students is critical for academic success. What does it mean? What makes it critical?

*Thank you for this comment. We adjusted this text, which is on lines 110-123 to clarify. It now indicates “* This study focuses on a diverse sample of first-year students. The first year is understood to be a particularly challenging year for students given the transition to a new school environment and the increased independence students experience [12,13].*” We also added citations to support the statement.*

2. In table 1, the authors listed the demographic characteristic among the two samples (cross-sectional and longitudinal). Why the authors did not include the family income as it mentioned earlier in the background as the related factors.

*We clarify on line 271 that free/reduced price lunch status is the proxy we have for low-income. This is the measure that is included in Table 1.*

3. Table 5 looks not clear. Why did the author provide three marginal effect with different values? Why did the sample in column 3,4,7,8 are different? What makes the difference? How did the authors deal with this issue?

*Thanks for pointing this out. Column 3(4) were marginal effects from a logistic regression on students without (with) moderate-severe anxiety symptoms in Wave I. Column 7(8) were marginal effects from a logistic regression on students without (with) moderate-severe depression symptoms in Wave I. We expected results to be different on these subsamples and discuss in the text how results differ for those with and without symptoms in Wave I. Please note that for formatting purposes these results are now split between Table 5 (first 4 columns from previous table with moderate-severe anxiety symptoms as the dependent variable) and Table 6 (last 4 columns from previous table with moderate-severe depression symptoms as the dependent variable), but reported marginal effects are the same.*

*We explain this more clearly now on lines 400 to 402. This reads:*

“The remaining regressions in Table 5 (6) were estimated separately for those with and those without Wave I anxiety (depression) symptoms and did not control for Wave I anxiety (depression) symptoms as a result.”

*We also add a row at the bottom of Tables 5 and 6 to indicate that the first two regressions were estimated on the whole sample. We also clarify the other row in the table that indicates whether the sample was estimated on the sample with or without Wave I symptoms. We changed the text from: “Moderate to severe symptoms in W1” to read now “Moderate to severe anxiety symptoms in Wave I?” and a similar row for Table 6, but replacing “anxiety” with “depression”.*

4. Still in table 5, why the authors were not able to calculate the odds ratio as this is important to find the likelihood to have high anxiety?

*We changed this so that we now report odds ratios for the regressions on the overall sample along with the marginal effects. Previously we just mentioned a preference for marginal effects given that it allows us to compare across model specifications.*

*We explain the logic now for the marginal effects in more detail on lines 402 to 413. It reads:*

“Odds ratios cannot be compared across model specifications when the sample or conditioning set changes, whereas marginal effects can be compared [43,44].”

*This is particularly important in our setting given that we want to compare how effect sizes change across different subsamples of our data and when we change the conditioning set.*

5. On page 18-19, the authors mentioned we found that students who were already experiencing mental health problems pre-pandemic were at greater odds of experiencing severe symptoms mid-pandemic.

*This is correct. In our models, we include a covariate for the students’ mental health pre-pandemic at Wave I. We now report both marginal effects (column 2) and odds ratios (column 1). The marginal effect of Wave I anxiety symptoms is 0.16 (Table 5) and the marginal effect of Wave I depression symptoms is 0.32 (Table 6). The corresponding odds ratios are 3.58 and 5.24 respectively.*

Minor comments:

Period should be placed after the citation. Please see the guideline.

*These are now fixed. Thanks for pointing out this error.*
